# Novel Magnetic Nanocomposites Based on Carboxyl-Functionalized SBA-15 Silica for Effective Dye Adsorption from Aqueous Solutions

**DOI:** 10.3390/nano12132247

**Published:** 2022-06-29

**Authors:** Claudia Maria Simonescu, Daniela Cristina Culita, Alina Tatarus, Teodora Mocanu, Gabriela Marinescu, Raul Augustin Mitran, Irina Atkinson, Andrei Kuncser, Nicolae Stanica

**Affiliations:** 1 Faculty of Chemical Engineering and Biotechnologies, Politehnica University of Bucharest, Polizu Street, No. 1-7, 011061 Bucharest, Romania; claudia.simonescu@upb.ro (C.M.S.); alina.tatarus@yahoo.com (A.T.); 2 Ilie Murgulescu Institute of Physical Chemistry, Romanian Academy, 202 Splaiul Independentei, 060021 Bucharest, Romania; tmocanu@icf.ro (T.M.); gmarinescu@icf.ro (G.M.); rmitran@icf.ro (R.A.M.); iatkinson@icf.ro (I.A.); nstanica@icf.ro (N.S.); 3 National Institute of Materials Physics, Atomistilor Street No. 405 A, 077125 Magurele, Romania; andrei.kuncser@infim.ro

**Keywords:** magnetite, carboxyl-functionalized SBA-15, adsorption, methylene blue, malachite green G

## Abstract

In this study, three novel magnetic nanocomposites based on carboxyl-functionalized SBA-15 silica and magnetite nanoparticles were prepared through an effective and simple procedure and applied for methylene blue (MB) and malachite green G (MG) adsorption from single and binary solutions. Structure, composition, morphology, magnetic, and textural properties of the composites were thoroughly investigated. The influence of the amount of carboxyl functional groups on the physicochemical and adsorptive properties of the final materials was investigated. The capacity of the synthesized composites to adsorb MB and MG from single and binary solutions and the factors affecting the adsorption process, such as contact time, solution pH, and dye concentration, were assessed. Kinetic modelling showed that the dye adsorption mechanism followed the pseudo-second-order kinetic model, indicating that adsorption was a chemically controlled multilayer process. The adsorption rate was simultaneously controlled by external film diffusion and intraparticle diffusion. It was evidenced that the molecular geometry of the dye molecule plays a major role in the adsorption process, with the planar geometry of the MB molecule favoring adsorption. The analysis of equilibrium data revealed the best description of MB adsorption behavior by the Langmuir isotherm model, whereas the Freundlich model described better the MG adsorption.

## 1. Introduction

One of the most serious environmental problems in modern society is water pollution, mainly due to effluents from textile, leather, paints and pigments, plastics, paper, food, and cosmetic industries [1]. Among the different pollutants, dyes represent one of the most problematic groups of organic pollutants due to their toxicity, which causes serious health effects on animals and human beings. Most of them are stable to biodegradation, photodegradation, and oxidizing agents. Some studies revealed their carcinogenic effects due to the changes induced in DNA synthesis [2,3]. The aquatic organisms are also affected by the presence of dyes in water because they hinder light penetration through the water surface decreasing the photosynthetic activity of phytoplankton. This causes oxygen deficiency and disturbs the biological cycle of aquatic biota [4]. Moreover, dye molecules in wastewater lead to mutagenicity, carcinogenicity, and the dysfunction of human beings’ kidney, liver, brain, reproductive system, and central nervous system [5]. The wastewater of dye industries is the maximum polluting place among all industrial segments, producing a huge volume of effluents. Textile industries yield tons of dyes that are discharged as wastewater every year throughout the dyeing procedures. Methylene blue (MB) and malachite green G (MG) are two of the most common cationic dyes, usually used in textiles, printing, leather, plastics, paints, pharmaceuticals, and food [6]. They are commonly used as coloring agents and antiseptics for external treatments on wounds and ulcers [6]. However, their oral consumption is poisonous and carcinogenic.

Therefore, the removal of dyes from water and wastewater is one of the most significant environmental problems. Over the years, several methods for the treatment of dye-containing wastewater have been studied: membrane separation, adsorption, photocatalysis, coagulation–flocculation, ion exchange, oxidation processes, electrochemical processes, etc. [7,8,9,10]. Among these techniques, adsorption is the most commonly used method owing to its high removal efficiency, versatility, simplicity, and low cost, using inexpensive and eco-friendly materials [11,12]. Various materials, such as activated carbon, zeolites, clays, nanoparticles, biomaterials, and metal oxides have been investigated for the removal of dyes from water [13]. In recent years, mesoporous silica particles have received a great interest in this field due to their special physicochemical properties, such as high surface areas and pore volumes, defined pore sizes, and ease of functionalization. SBA-15 is one of the most known examples of mesoporous silica. Its efficiency in adsorption processes is due to its large internal surface and mesoporous structure which allows facile access of molecules. Moreover, in order to increase its adsorption capacity and specificity for certain pollutants, SBA-15 can be easily functionalized due to the high reactivity and concentration of silanol groups (Si–OH) on its surface. The literature data showed that various functional groups, including NH_2_, Cl, SH, CN, SO_3_H, phenyl, vinyl, etc., may be grafted on the surface of SBA-15 [14,15,16].

Despite the advantages offered by SBA-15 mesoporous silica, it has the disadvantage of the difficulty of separation from the suspension after the completion of the adsorption process. The use of magnetic separation is an efficient and modern solution to counteract this drawback. The magnetic adsorbents have gained significantly increased interest for environmental applications owing to their high efficiency, ease of separation after adsorption by applying an appropriate magnetic field, high mechanical and chemical stability, tunable chemical composition and morphology, and fast recovery. The modification of a mesoporous silica-based adsorbent by inclusion of a magnetic component allows easier separation after adsorption, compared to centrifugation or filtration. This type of magnetic mesoporous silica structure offers a large accessible surface area and pore volume for the fast adsorption of various molecules. Several groups have reported the synthesis of mesoporous silica microspheres embedding magnetic particles utilizing various template agents and used these materials as magnetically separable adsorbents for specific dissolved pollutants [17,18]. Among the organic functionalities that can be attached to the silica surface, carboxyl groups are particularly interesting for adsorbing contaminants. This exceptional adsorption capacity is owing to an increase in their negative charge density in neutral or basic aqueous conditions, which leads to the formation of particular binding sites for adsorbates via deprotonation [19]. To the best of our knowledge, there are only very few studies regarding the adsorption of cationic dyes on magnetic mesoporous silica functionalized with carboxylic groups. Fu et al. reported superparamagnetic mesoporous silica microspheres embedded with iron oxide particle cores uniformly functionalized with carboxylic groups for removing methylene blue and acridine orange from water. They used stearyl trimethyl ammonium bromide as a surfactant template [20]. The obtaining of carboxyl-functionalized mesoporous silica materials is usually based on the hydrolysis of cyanide-modified silica with sulfuric acid [21,22]. These conditions of hydrolysis severely limit the obtaining of carboxyl-functionalized mesoporous silica materials embedded with iron oxide particles’ magnetic core.

**Figure 1:**
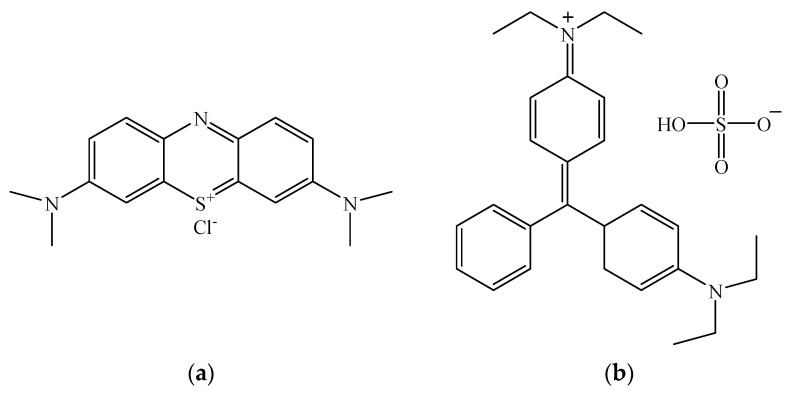
Chemical structure of (**a**) Methylene Blue and (**b**) Malachite Green G.

The aim of this paper was to obtain magnetic nanocomposites based on carboxyl-functionalized SBA-15 silica and magnetite nanoparticles through a convenient and effective procedure and to evaluate their effectiveness as adsorbents for cationic dyes. The influence of the amount of carboxyl functional groups on the physicochemical and adsorptive properties of the final nanomaterials was investigated. All the nanocomposites were characterized by nitrogen adsorption–desorption analysis, Fourier-transform infrared (FT-IR) spectroscopy, thermogravimetric (TGA) and elemental analysis, zeta potential, X-ray diffraction (XRD), scanning/transmission electron microscopy (SEM/TEM), and the vibrating-sample magnetometer technique (VSM). Methylene blue (MB) and malachite green G (MG) (Figure 1) were used as model pollutants in the adsorption tests. The adsorption process was investigated as a function of solution pH, contact time, and initial concentration of dye. The characteristics of the adsorption isotherms and kinetics were also studied in single and binary solutions.

## 2. Materials and Methods

### 2.1. Materials

Triblock copolymer Pluronic P-123 (Poly(ethylene glycol)-block-poly(propylene glycol)-block-poly(ethylene glycol)) average M_n_ ~5800 (Sigma-Aldrich, Steinheim, Germany), (2-Cyanoethyl)triethoxysilane 97% (CTES) (Alfa Aesar, Karlsruhe, Germany), tetraethoxysilane > 99% (TEOS), ferric chloride hexahydrate > 99% (FeCl_3_·6H_2_O), ferrous chloride tetrahydrate > 98% (FeCl_2_·4H_2_O), sulfuric acid 95–97% (H_2_SO_4_), hydrochloric acid 2 M (HCl) (Merck, Darmstadt, Germany), ammonia solution (NH_4_OH 25 wt.%) (Chimreactiv, Bucharest, Romania), Methylene Blue Reag. Ph Eur (C_16_H_18_ClN_3_S·3H_2_O), and Malachite Green G analytical standard (C_27_H_34_N_2_O_4_S) (Sigma-Aldrich, Steinheim, Germany) were commercial reagent grade.

### 2.2. Synthesis of Magnetic Nanocomposites Based on Carboxyl-Functionalized SBA-15 Silica

3.0 g of P-123 was dissolved in 93 mL hydrochloric acid 2M solution until the solution became clear. 2-Cyanoethyltriethoxysilane was then added and the solution was stirred at 40 °C for 30 min. The mixture was transferred to a polypropylene bottle, then TEOS was slowly added and the mixture was kept under stirring at 40 °C for 22 h, followed by aging in an oven at 100 °C for 45 h, under static conditions. The solid product was separated by centrifugation, washed several times with distilled water and then dried at 90 °C in oven.

The molar composition of the mixture was (1 − x) TEOS: x CTES: 6 HCl: 167 H_2_O: 0.017 P123, where x = 0, 0.05, and 0.1. For the hydrolysis of the –CN groups, the product was treated with 150 mL 48 wt.% H_2_SO_4_ at 95 °C for 24 h [21,22]. Subsequently, the mixture was filtered on a G4 fritted filter funnel and the solid product was washed with a copious amount of water until the eluent became neutral, then several times with hot ethanol. The obtained carboxyl-functionalized mesoporous silica samples were denoted: SBA15-COOH-x, where x = 0, 0.05, and 0.1.

The magnetite particles were prepared according to a previous paper [23]. To an aqueous solution of 2.77 mmol FeCl_3_·6H_2_O and 1.38 mmol FeCl_2_·4H_2_O in 60 mL deionized water, 30 mL of 25% NH_4_OH solution was added under vigorous stirring. After heating at 80 °C for one hour, the resulting black precipitate was separated with the aid of a neodymium magnet and washed with distilled water until the pH of the runoff was neutral. The as-prepared magnetite particles were dispersed in water/ethanol mixture (volume ratio 4:3) by sonication; then, 1.2 mL of 25% NH_4_OH solution and 0.3 mL TEOS were consecutively added to the suspension; then, the mixture was sonicated 20 min. To this mixture, a certain amount of SBA15-COOHx dispersed in water/ethanol (volume ratio 4:3) was added under strong stirring. The mixture was sonicated for 45 min, then stirred continuously for 20 h at room temperature. The obtained solid materials were separated by centrifugation, washed three times with water, and dried in air. The final magnetic nanocomposites were denoted Fe_3_O_4_-SBA15, Fe_3_O_4_-SBA15-COOH-0.05, and Fe_3_O_4_-SBA15-COOH-0.1.

### 2.3. Characterization Methods

The nitrogen adsorption–desorption isotherms at −196 °C were measured using a Micromeritics ASAP 2020 analyzer (Norcross, GA, USA). The samples were degassed at 100 °C for 6 h under vacuum before analysis. Specific surface areas (S_BET_) were estimated according to Brunauer–Emmett–Teller (BET) method, using adsorption data in the relative pressure range 0.05–0.30. The total pore volume (V_total_) was calculated from the amount adsorbed at the relative pressure of 0.99. The average pore diameter and pore size distribution curves were obtained using Barrett–Joyner–Halenda (BJH) method using the desorption branch. FT-IR spectra were obtained on a Jasco FT/IR-4700 spectrophotometer (Tokyo, Japan) using KBr pellets. Thermogravimetric analyses (TGA) were performed using a Mettler Toledo TGA/SDTA851e thermogravimeter (Mettler Toledo, Greifensee, Switzerland), using a heating rate of 10 °C min^−1^, open alumina crucibles, and a 20 mL min^−1^ synthetic air flow. The content of C, H, and N was determined through elemental analysis on a EuroEA elemental analyzer (HEKAtech GmbH, Wegberg, Germany). Zeta potential was measured by electrophoretic light scattering using a Backman Coulter Delsa Nano C particle analyzer (Brea, CA, USA) with 100 μg/mL sample dispersed in distilled water. Samples were ultrasonicated for 15 min before measurement. The powder X-Ray diffraction (XRD) analysis was performed on a Rigaku Ultima IV diffractometer (Rigaku Co., Tokyo, Japan) using a monochromatic Cu Kα (λ = 1.5418 Å) radiation source operated at 40 kV and 30 mA. The wide-angle diffractograms were recorded in the 2θ range 10–80°, with 2° min^−1^ scan speed and 0.02° step width, while the low-angle diffractograms were recorded between 0.6 and 5°, with 1° min^−1^ scan speed and 0.02° step width. XRD data were analyzed using Rigaku’s PDXL software connected to ICDD PDF-2 database. The average crystallite size was calculated using the Williamson–Hall method. Microstructural analysis was performed on a TESCAN LYRA 3 XMU scanning electron microscope (SEM) (Tescan Orsay Holding, Brno-Kohoutovice, Czech Republic). JEOL 2100 electron microscope equipped with LaB6 filament and high-resolution polar piece (JEOL GmbH, Freising, Germany) was used for transmission electron microscopy (TEM) investigations. The magnetic properties were measured at room temperature on a Lake Shore’s fully integrated Vibrating-Sample Magnetometer system 7404 (VSM) (Westerville, OH, USA). The experimental data were analyzed by fitting to the Langevin function.

### 2.4. Adsorption Experiments

The effect of pH, time, and concentration of dye solution on the adsorption capacity of the samples was studied via batch experiments in 100 mL conical flasks containing 5 mg adsorbent/20 mL of each of the dye solutions. All mixtures were stirred at 150 rpm on a GFL 3015 orbital shaker (Burgwedel, Germany). Stock solutions of dyes were obtained by dissolving 1 g of the respective dye in 1 L of distilled water, followed by dilution to obtain the desired concentration: between 5 and 100 mg/L. HCl and NH_4_OH solutions of various concentrations were used for dye solution’s pH changing. The pH of the dye solution was determined at room temperature using an Agilent 3200 laboratory pH meter (Agilent Technologies, Shanghai, China). After achieving equilibrium, the dye-loaded adsorbents were separated using a hand-held magnet and the dye concentration in the remaining solutions was determined. MB and MG analysis was performed on an Agilent 1200 series HPLC (Tokyo, Japan) system equipped with: semipermeable membrane degasser, quaternary pump, autosampler with variable injection volume (0.1–100 µL), thermostatted column compartment, and a diode array detector (DAD) with the ability to record simultaneously UV-VIS spectra (190–900 nm) and up to 8 discrete wavelengths in this range. All chromatographic runs were carried out on an Acclaim Surfactant Plus column (150 × 3.0 mm, 3.0 µm) from Thermo Scientific. The detection of the target compounds was performed at the absorption maximum of λ = 665 nm (MB) and λ = 610 nm (MG) which were observed in the UV-Vis spectrum obtained by HPLC-DAD. Agilent ChemStation software was used for data acquisition, processing, and reporting.

The adsorption capacity was calculated using the following formula:

(1) $$Q_{e}=\frac{\left(C_{i}-C_{e}\right)\cdot V}{m}$$

where *Q_e_*—the amount of dye adsorbed at equilibrium (mg g^−1^), *C_i_*—the initial concentration of dye solution (mg L^−1^), *C_e_*—the equilibrium concentration of dye solution (mg L^−1^), *V*—total volume of dye solution (L), and *m*—mass of adsorbent used (g).

All the adsorption tests were performed in triplicate with a maximum experimental error of 5%.

## 3. Results and Discussion

### 3.1. Materials Characterization

**Figure 2:**
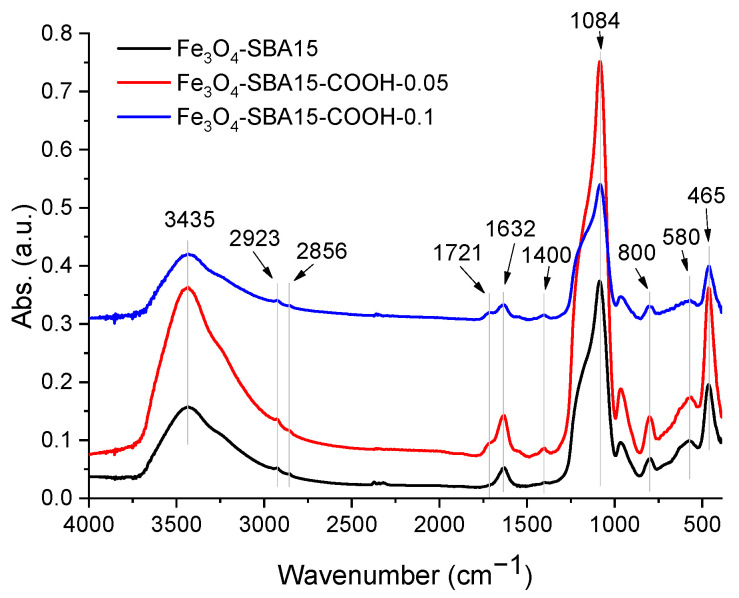
FTIR spectra of Fe_3_O_4_-SBA15, Fe_3_O_4_-SBA15-COOH-0.05, and Fe_3_O_4_-SBA15-COOH-0.1.

The FTIR spectra of magnetite-containing samples are shown in Figure 2. The band corresponding to the stretching vibration of the C=O bond of carboxyl groups, at 1721 cm^−1^, is clearly evidenced in the spectra of Fe_3_O_4_-SBA15-COOH-0.05 and Fe_3_O_4_-SBA15-COOH-0.1 and is absent in the spectrum of Fe_3_O_4_-SBA15. The appearance of this band and the absence of that corresponding to CN-stretching vibration (2252 cm^−1^) confirms the hydrolysis of all cyano groups [21,22]. A close inspection of this band revealed that its intensity increases with the content of CTES in the silica source. This confirms that the amount of carboxyl groups in Fe_3_O_4_-SBA15-COOH-0.1 is higher than in Fe_3_O_4_-SBA15-COOH-0.05. This aspect is much more evident in the spectra of carboxyl-functionalized SBA-15 silica samples without magnetite content (Figure S1). Moreover, a slight displacement of this band from 1717 to 1721 cm^−1^ is observed in the case of magnetic nanocomposites compared to the corresponding samples of carboxyl-functionalized silica. This shift could be attributed to the interactions of carboxyl groups with the surface of magnetite nanoparticles. The bands at 1084 and 800 cm^−1^ correspond to asymmetric and symmetric stretching vibration of Si-O-Si, while that at 465 cm^−1^ to asymmetric deformation vibration of O-Si-O [24]. The stretching vibrations of hydroxyl groups on the silica surface and those of adsorbed water appear at 1632 and 3435 cm^−1^. The bands at 2923 and 2856 cm^−1^ are assigned to asymmetric and symmetric vibrations of C-H bonds in the ethyl chain anchored on the silica surface. The presence of magnetite nanoparticles was confirmed by the absorption band at 580 cm^−1^ which corresponds to the Fe–O bond [25].

**Figure 3:**
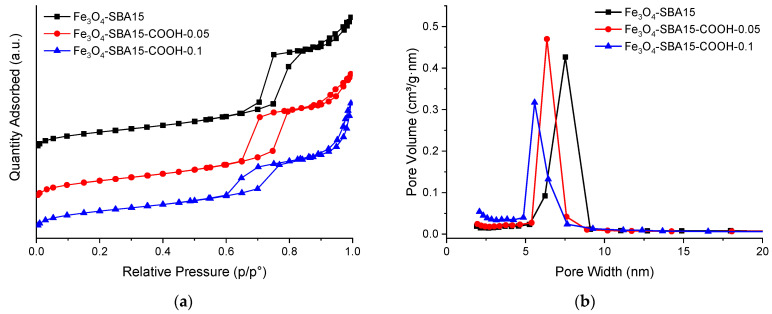
Nitrogen adsorption–desorption isotherms (**a**) and pore size distribution curves (**b**) of Fe_3_O_4_-SBA15 (black line), Fe_3_O_4_-SBA15-COOH-0.05 (red line), and Fe_3_O_4_-SBA15-COOH-0.1 (blue line).

Figure 3 shows the nitrogen adsorption–desorption isotherms and pore size distribution curves of the magnetic nanocomposites. All these samples exhibit type IV isotherms with an H1 type hysteresis characteristic for materials with ordered mesoporous structure, according to IUPAC classification [26]. One can notice that the CTES molar content in the total silica source has a deep effect on the formation of SBA-15 ordered mesostructure. As the CTES content increases, the hysteresis closure point shifts slightly to lower *p*/*p*° values, while the desorption branch becomes less steep, indicating a decrease in average pore diameters. This observation is also confirmed by the pore size distribution (PSD) calculated by the BJH method (Figure 3, right side). The increasing of CTES content also leads to the decrease in specific surface area of the carboxylated-SBA-15 samples compared with pure SBA15, which proves the immobilization of carboxyl functional groups on the internal surface of mesoporous silica channels (Table 1). Judging from the shape of the hysteresis loop, it is clear that the introduction of a larger amount of CTES into the synthesis will lead to the destruction of the uniform mesoporous structure. The magnetic nanocomposites have lower surface areas and pore volumes than carboxylated-SBA15 samples, but close to each other, while the PSD curves are similar to those of the corresponding carboxylated-SBA15 materials (Figure S2). This is an indication that the mesoporous silica interacted with the magnetite nanoparticles mainly on the external surface through the carboxyl groups which facilitated the interaction and to a lesser extent at the entrance of the pores.

**Figure 4:**
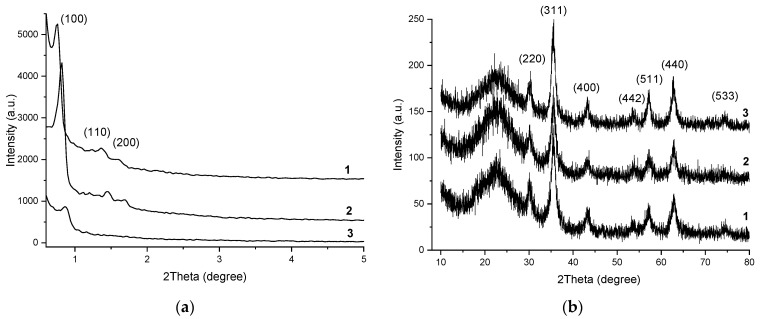
Low-angle (**a**) and wide-angle (**b**) XRD patterns of the samples Fe_3_O_4_-SBA15 (**1**), Fe_3_O_4_-SBA15-COOH-0.05 (**2**), and Fe_3_O_4_-SBA15-COOH-0.1 (**3**).

Figure 4a shows the low-angle XRD powder diffraction patterns of the samples. Three well-resolved peaks which can be indexed as (100), (110), and (200) diffraction peaks associated with P6mm hexagonal symmetry of SBA-15 can be observed for Fe_3_O_4_-SBA15 and Fe_3_O_4_-SBA15-COOH-0.05 [27]. This suggests a highly ordered hexagonal structure of the SBA15 silica in these two samples. For the sample Fe_3_O_4_-SBA15-COOH-0.1, the intensity of the diffraction peaks decreased compared with the other two samples, indicating that the increasing of the content of COOH functional groups induces a deviation from the relatively ordered structure of the SBA15. Figure 4b shows the wide-angle XRD powder diffraction patterns of the samples. The reflections at 2theta: 29.98(220), 35.58(311), 43.05(400), 53.48(442), 57.14(511), 62.92(440), and 74.30(533) values, confirm the presence of magnetite with an inverse cubic spinel structure in the investigated samples [28]. The crystallite size of the magnetite was calculated to be ~6 nm.

**Figure 5:**
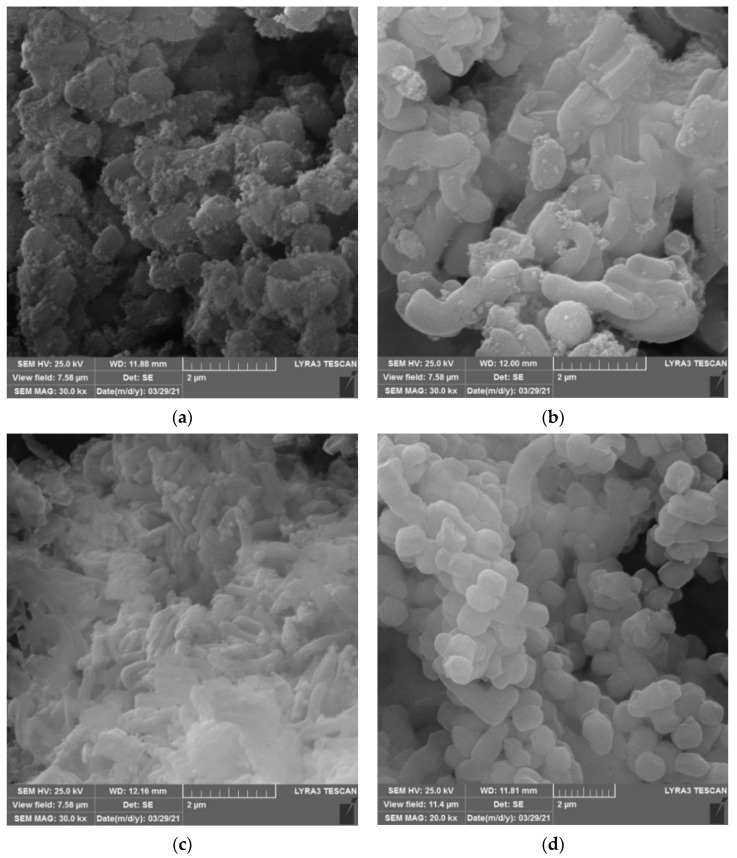
SEM images of Fe_3_O_4_-SBA15 (**a**), Fe_3_O_4_-SBA15-COOH-0.05 (**b**), Fe_3_O_4_-SBA15-COOH-0.1 (**c**), and SBA-15 (**d**).

SEM micrographs of Fe_3_O_4_-SBA15, Fe_3_O_4_-SBA15-COOH-0.05, and Fe_3_O_4_-SBA15-COOH-0.1 (Figure 5a–c) show quasi-spherical magnetite nanoparticles anchored on the surface of carboxyl-modified worm-like SBA-15 particles. Comparing the morphology of carboxyl-functionalized SBA-15 particles in magnetic composites with that of bare SBA-15 (Figure 5d), one can notice an elongation and a thinning of the filaments with the increase in -COOH content. The most pronounced morphological change is observed in the case of Fe_3_O_4_-SBA15-COOH-0.1 in which the amount of -COOH groups is the highest (Figure 5c). However, the worm-like SBA-15 particles included in the magnetic composites have shorter one-dimensional pore channels which could facilitate mass diffusion within the pore channels.

**Figure 6:**
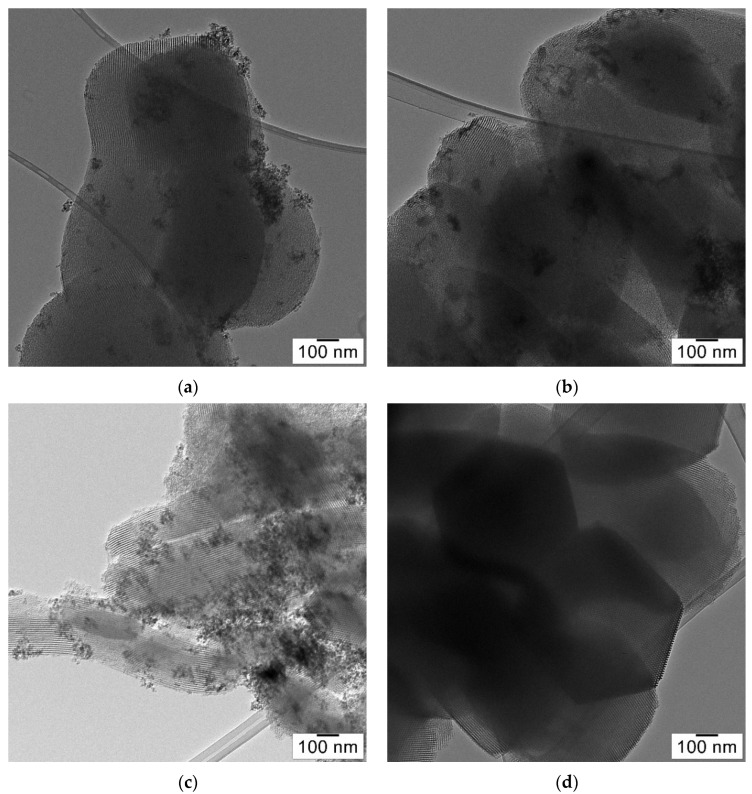
TEM images of Fe_3_O_4_-SBA15 (**a**), Fe_3_O_4_-SBA15-COOH-0.05 (**b**), Fe_3_O_4_-SBA15-COOH-0.1 (**c**), and SBA-15 (**d**).

**Figure 7:**
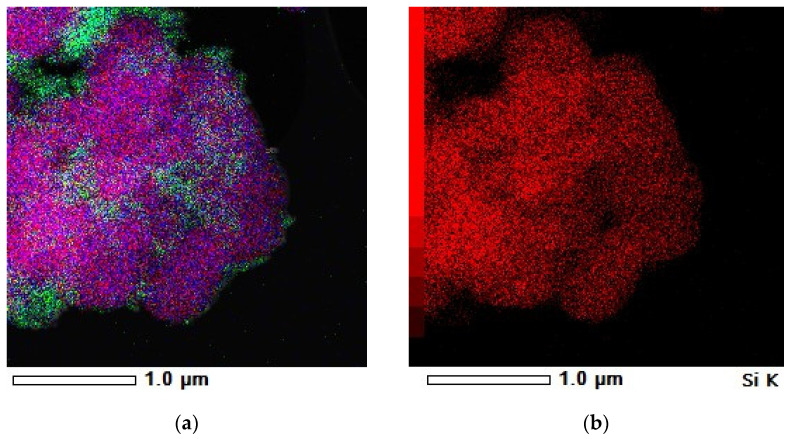

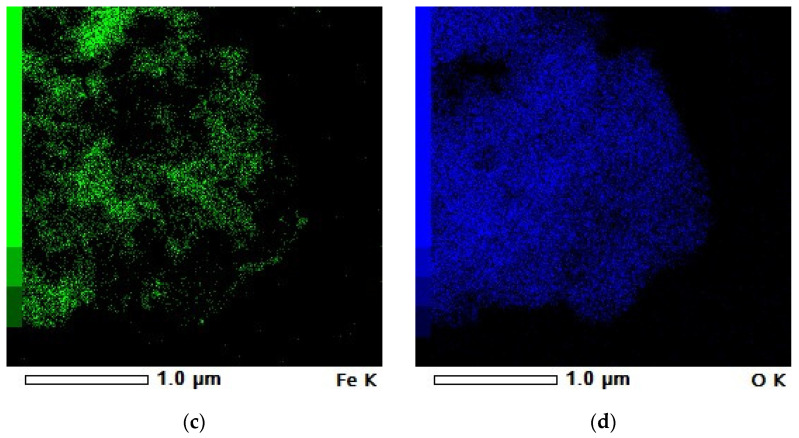
Compositional elemental mapping of Fe_3_O_4_−SBA15: (**a**) all elements; (**b**–**d**) the corresponding elemental mapping of Si, Fe, and O, respectively.

Transmission electron microscopy (TEM) images of the magnetic composites and bare SBA-15 are shown in Figure 6. All TEM images clearly display the parallel arrangement of hexagonal pore channels of ~8 nm, characteristic of SBA-15 silica. It can be noticed that the highly ordered mesoporous structure of SBA-15 was preserved regardless of the COOH content. The dark small spots of ~8 nm represent the magnetite nanoparticles, randomly distributed on the SBA-15 surface. The elemental mapping shown in Figure 7 only for Fe_3_O_4_-SBA15 shows the homogeneous distribution of Fe_3_O_4_ nanoparticles onto the surface of SBA-15.

**Figure 8:**
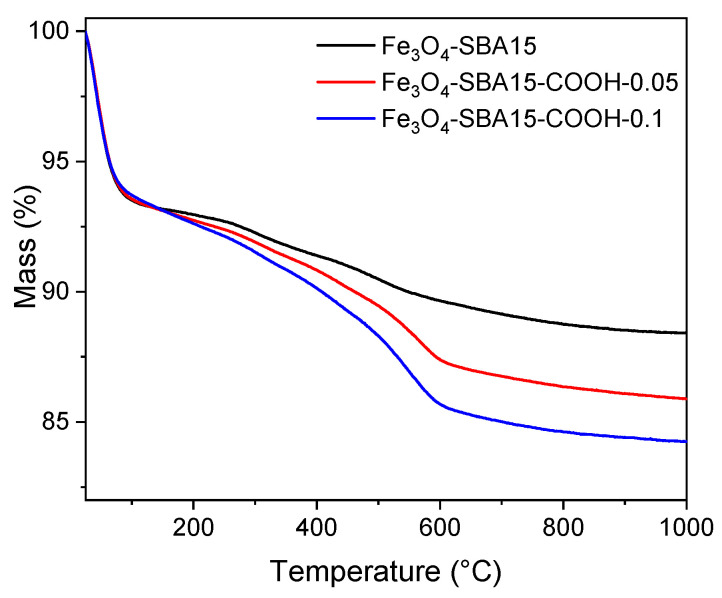
TG curves of Fe_3_O_4_-SBA15, Fe_3_O_4_-SBA15-COOH-0.05, and Fe_3_O_4_-SBA15-COOH-0.1.

Thermogravimetric analyses (TGA) were carried out in order to investigate the thermal behavior and composition of the magnetic nanocomposites. The loss of physisorbed water can be noticed for all materials on heating up from 25 to 140 °C (Figure 8). Two superimposed mass loss events can be noticed in the temperature range 140–650 °C for all the samples. These two thermal events roughly centered at 300 and 500 °C correspond to the combustion of organic groups and the oxidation of magnetite to Fe_2_O_3_. The oxidation of Fe_3_O_4_ to Fe_2_O_3_ after the heat treatment was confirmed visually, as the materials changed color from brown-black before the TG analysis to the characteristic red color of Fe_2_O_3_. The mass decrease up to 650 °C corresponds to 4.1, 6.6, and 8.5% wt. for the Fe_3_O_4_-SBA15, Fe_3_O_4_-SBA15-COOH-0.05, and Fe_3_O_4_-SBA15-COOH-0.1 samples, respectively. All three samples exhibit a gradual, 1.1% mass loss above 650 °C, which can be ascribed to the dehydration of silanol groups [29]. The percent of organic material was calculated by subtracting the data obtained by TGA for the carboxyl-containing samples and Fe_3_O_4_-SBA15. The values obtained showed a percentage of organic component of 1.8% for Fe_3_O_4_-SBA15-COOH-0.05 and 4.2% for Fe_3_O_4_-SBA15-COOH-0.1. Both values agree well with those obtained by elemental analysis (Fe_3_O_4_-SBA15 exp. H 3.48%; Fe_3_O_4_-SBA15-COOH-0.05 exp. C 2.74%, H 4.49%; Fe_3_O_4_-SBA15-COOH-0.1 exp. C 3.59%, H 4.72%).

**Figure 9:**
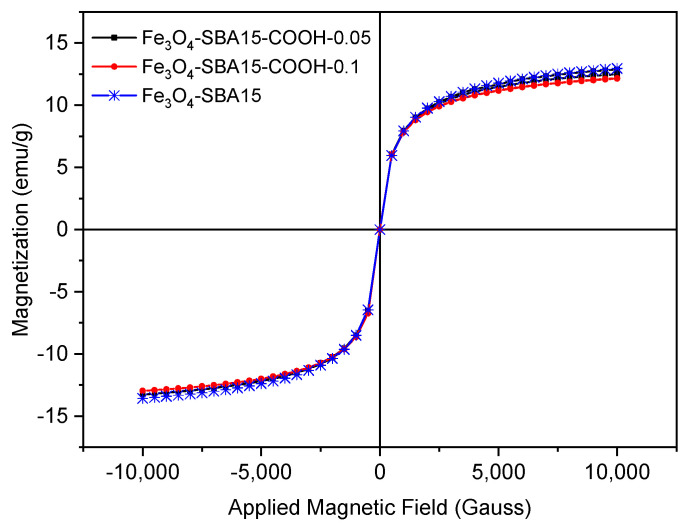
Magnetization curves of Fe_3_O_4_-SBA15, Fe_3_O_4_-SBA15-COOH-0.05, and Fe_3_O_4_-SBA15-COOH-0.1 at room temperature.

The magnetic properties of the samples were investigated using a vibrating-sample magnetometer (VSM) at room temperature. The magnetization curves (Figure 9) indicate a superparamagnetic behavior of all samples. The calculated saturation magnetization values are similar: 13.3 emu/g for Fe_3_O_4_-SBA15, 13.0 emu/g for Fe_3_O_4_-SBA15-COOH-0.05, and 12.5 emu/g for Fe_3_O_4_-SBA15-COOH-0.1. The Ms values are smaller than that of bulk Fe_3_O_4_ (88–94 emu/g) due to the presence of the diamagnetic silica particles. However, these values indicate that the investigated nanocomposites can be isolated quickly and efficiently from solution under the influence of an external magnetic field. For the adsorption processes of pollutants from aqueous solutions, this property is particularly important.

### 3.2. Adsorption Studies

#### 3.2.1. Effect of pH on Dye Adsorption

**Figure 10:**
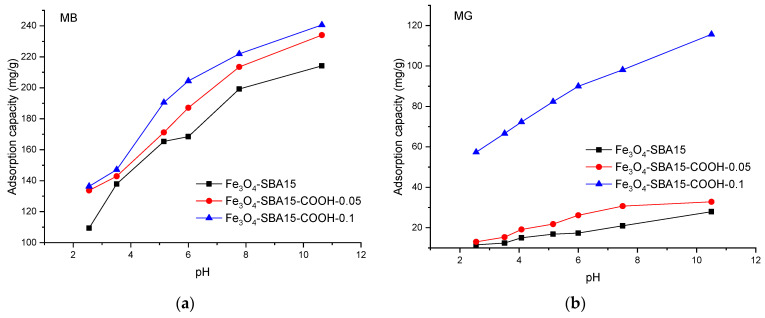
Effect of pH on adsorption capacity of the samples for MB (**a**) and MG (**b**) from single solutions.

The solution pH is a crucial factor for the adsorption process because it affects both the surface of the adsorbent and the adsorbate structural changes. The effect of pH on dye adsorption was studied in the range 2–11. According to the obtained results (Figure 10), in both cases the adsorption capacities increase by increasing the pH and reach a maximum at pH 10.6. Consequently, this value was selected for the following investigations. Low removal efficiency in acidic solutions can be explained by the fact that at low pH values, the carboxyl groups are protonated; hence, the interaction with the cationic dyes’ molecules is lower. As the pH increases, more and more carboxyl functional groups dissociate and the surface of the adsorbent becomes more negative; therefore, the electrostatic attraction between the adsorbent and dye molecules increases.

#### 3.2.2. Effect of Contact Time on Dye Adsorption

**Figure 11:**
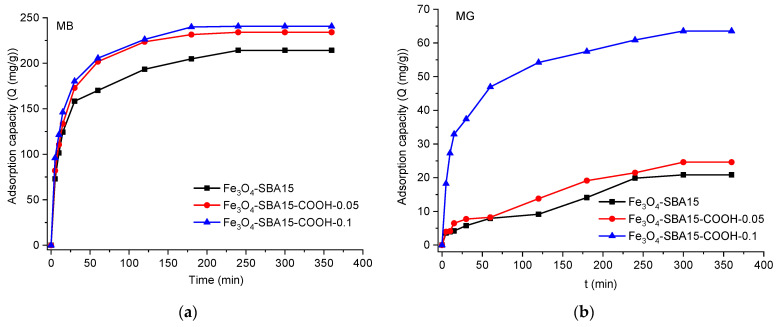
Variation in adsorption capacity versus contact time at pH = 10.6 for MB (**a**) and MG (**b**).

The adsorption of MB onto all three adsorbents is a rapid process, with the adsorption rate increasing quickly during the first 30 min, then slowing down gradually with time until reaching the equilibrium (after 240 min) (Figure 11). This behavior can be explained as follows: during the early stage of adsorption, the number of active sites available on the external and internal surface of the adsorbent and the adsorbate concentration gradient is high, leading to a fast adsorption rate. In the second stage, when the most accessible surface sites tend to saturate, MB gradually diffuses to the active sites located on the internal surface of the mesoporous silica, in the smallest pores; hence, the adsorption rate decreases. After 240 min, the concentration of the MB solution remains almost unchanged, indicating that all the adsorption sites were saturated. In the case of MG, Fe_3_O_4_-SBA15-COOH-0.1 behaves similar as for MB, while for Fe_3_O_4_-SBA15 and Fe_3_O_4_-SBA15-COOH-0.05, the adsorption rate increases monotonically from the beginning of the process until the equilibrium (after 300 min).

#### 3.2.3. Adsorption Kinetics

The kinetics of the adsorption process is essential to understand the adsorption mechanism and for optimization of the operating conditions in full-scale batch processes. The nonlinear forms of all these kinetic models are described by the following equations:

- Pseudo-first-order model:

(2) $$Q_{t}=Q_{e}\left(1-e^{-k_{1}t}\right)$$

- Pseudo-second-order model:

(3) $$Q_{t}=\frac{Q_{e}^{2}k_{2}t}{\;1+Q_{e}k_{2}t}$$

- Intraparticle diffusion model:

(4) $$Q_{t}=k_{id}t^{0.5}+C$$

where *Q_e_* and *Q_t_* are the amount of dye adsorbed at equilibrium and at time *t* (mg g^−1^), *k*_1_ is the rate constant of pseudo-first-order kinetics (min^−1^), *k*_2_ is the rate constant of pseudo-second-order kinetics (g mg^−1^ min^−1^), *k_id_* is the intraparticle diffusion rate constant (g mg^−1^ min^−1^) and the intercept of the plot, and *C* reflects the boundary layer effect.

**Figure 12:**
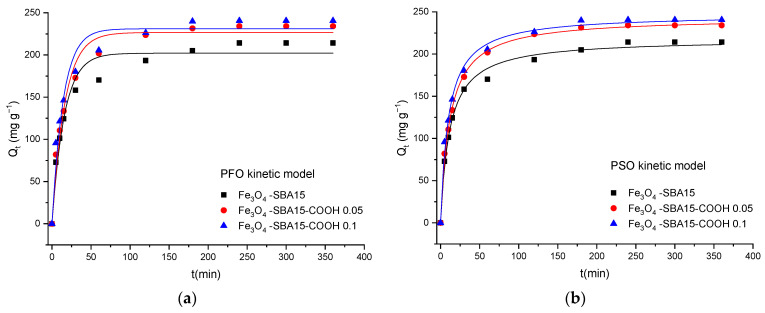
Graphical representation of the PFO (**a**) and PSO (**b**) kinetic models for adsorption of MB onto magnetic nanocomposites from single solutions (nonlinear regression).

**Figure 13:**
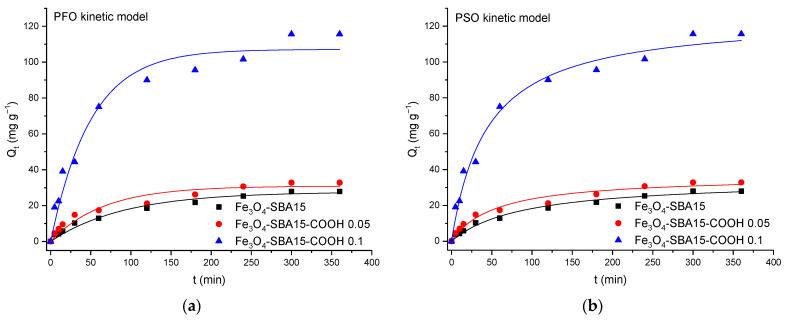
Graphical representation of the PFO (**a**) and PSO (**b**) kinetic models for adsorption of MG onto magnetic nanocomposites from single solutions (nonlinear regression).

**Figure 14:**
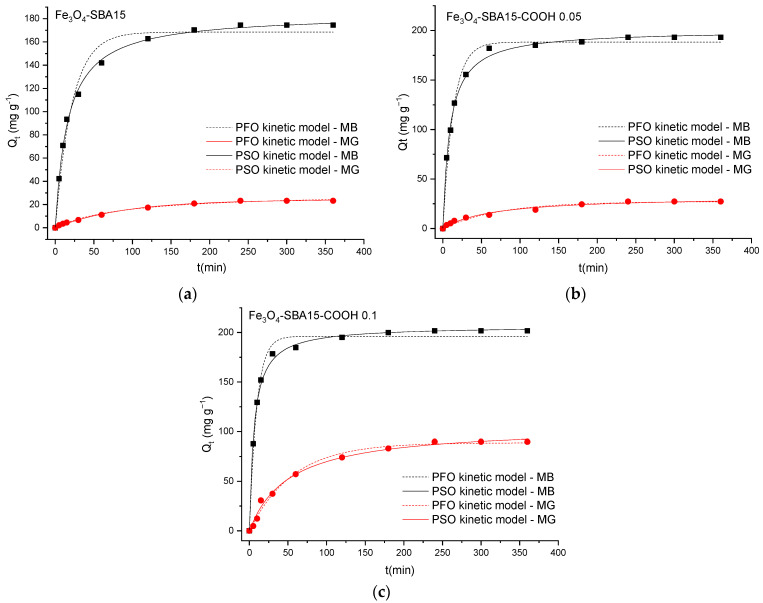
Graphical representation of the PFO and PSO kinetic models for competitive adsorption of MB and MG onto Fe_3_O_4_−SBA15 (**a**), Fe_3_O_4_−SBA15−COOH−0.05 (**b**), and Fe_3_O_4_−SBA15-COOH−0.1 (**c**) from binary solutions (nonlinear regression).

Figure 12, Figure 13 and Figure 14 show the PFO and PSO kinetic models fitting the data for MB and MG adsorption onto magnetic composites in single and binary solutions, while Table 2 and Table 3 display the values of the calculated kinetic parameters using the PFO and PSO nonlinear models. As can be seen, the values of adjusted R^2^ are higher for the PSO model than for the PFO model in all the cases, indicating that the PSO model is more suitable to describe the adsorption process of MB and MG, respectively, onto the magnetic nanocomposites. In addition, the theoretical values of *Q_e_* (*Q_e cal_*) correlate better with the experimental ones (*Q_e exp_*), confirming that the adsorption process follows a PSO kinetic model and chemisorption is the rate-limiting step [30].

**Figure 15:**
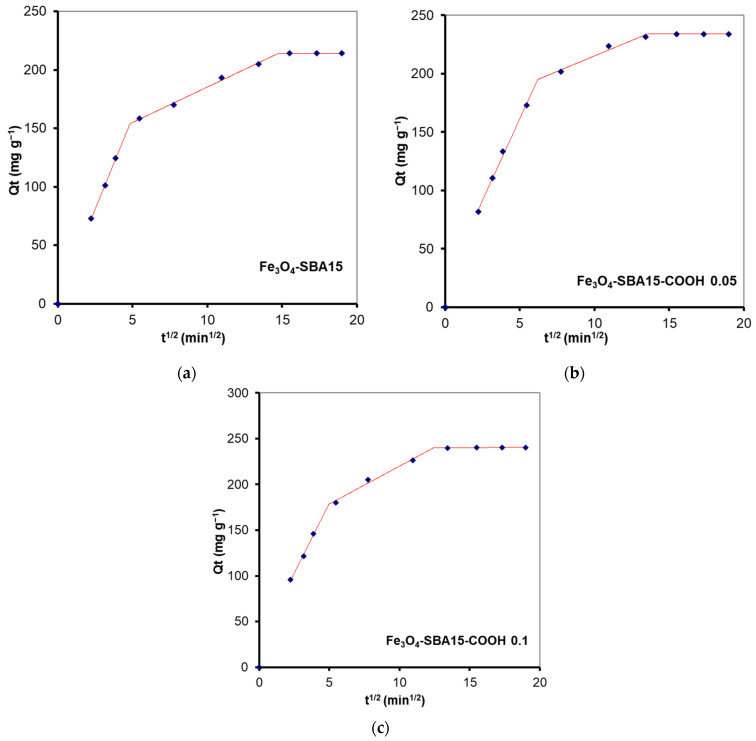
Kinetic modelling of the experimental data obtained from the adsorption process of MB onto Fe_3_O_4_−SBA15 (**a**), Fe_3_O_4_−SBA15−COOH−0.05 (**b**), and Fe_3_O_4_−SBA15-COOH−0.1 (**c**) using the intraparticle diffusion model.

The phenomena that limit the sorption mechanism are usually determined using the Weber and Morris kinetic intraparticle diffusion model. According to this model, if a straight line passing through the origin is generated from the plot of Equation (4), it can be said that the adsorption mechanism involves intraparticle diffusion of the species [31]. The slope of the linear curve is the rate constant of the intraparticle diffusion process. In our study, when the kinetic data obtained for MB adsorption onto the magnetic composites were analyzed using the Weber and Morris intraparticle diffusion model, it was observed that the plot did not pass through the origin, indicating that intraparticle diffusion was not the only rate-limiting step. According to this model, the adsorption of MB occurs in three stages revealed through a multilinear curve composed of three segments (Figure 15). In this case, a piecewise linear regression was applied to the experimental data using a Microsoft Excel worksheet developed by Malash and El-Khaiary [32]. The results are presented in Figure 15 and Table 4. In the first stage, the adsorption is limited to the external diffusion of the adsorbate. The second stage consists of gradual adsorption of the adsorbate being limited by intraparticle diffusion, while the third one corresponds to an equilibrium phase during which the adsorption capacity remains stable [33]. This pattern shows that both external mass transfer and intraparticle diffusion are involved in adsorption.

**Figure 16:**
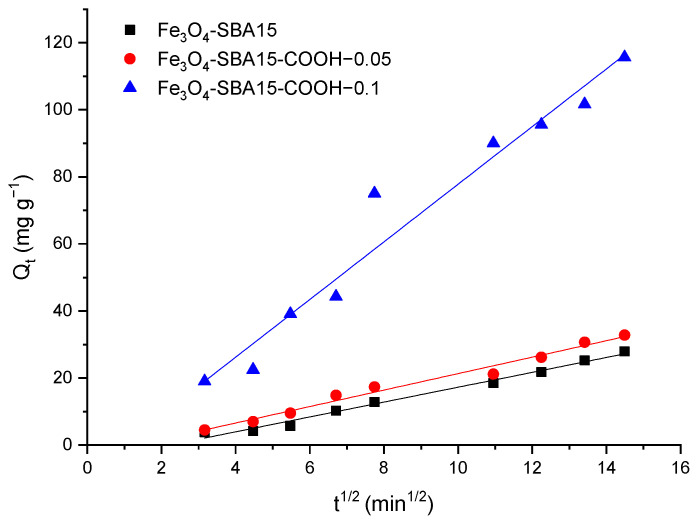
Intraparticle diffusion plots for the adsorption of MG onto magnetic nanocomposites.

In the case of MG adsorption, the variation in *Q_t_* versus t^0.5^ is shown in Figure 16. The slope of the straight line (*k_id_*), the intercept (*C*), and the regression coefficients are given in Table 4. It can be observed that the straight line did not pass through the origin which means that intraparticle diffusion is not the only rate-limiting step and the intercept has negative values. Previous studies that reported a negative value of *C* suggested that external film diffusion, in addition to intraparticle diffusion, limited the adsorption rate [34]. Therefore, in our case, the negative values of *C* constant might be interpreted as an external film diffusion resistance that led to the time lag for the MG adsorption [35].

#### 3.2.4. Adsorption Isotherms

The Langmuir (Equation (5)) and Freundlich (Equation (6)) isotherm models are the most known isotherm models that can describe the adsorption equilibrium between the concentration of the dye in bulk solution and the quantity of the dye adsorbed on the adsorbent surface at a given temperature. The Langmuir model assumes uniform adsorption energies distributed on the adsorbent surface, whereas the Freundlich isotherm model assumes multilayer adsorption on a heterogeneous surface with nonuniform affinities and adsorption energies [36]. In our study, the equilibrium data were modelled using both equilibrium isotherm models whose nonlinear equations are the following:

(5) $$Q_{e}=\frac{Q_{max}K_{L}C_{e}}{1+K_{L}C_{e}}$$

(6) $$Q_{e}=K_{F}\times C_{e}^{\frac{1}{n}}$$

where *C_e_* is the equilibrium concentration of the solute in solution (mg L^−1^), *K_L_* is the equilibrium constant of the Langmuir model related to the adsorption energy (L mg^−1^), *Q_e_* is the adsorption capacity at equilibrium (mg g^−1^), *Q_max_* is the maximum adsorption capacity (mg g^−1^), and *K_F_* and 1/*n* are Freundlich isotherm parameters (adsorption capacity (mg g^−1^) and intensity).

Figures S3 and S4 and Table 5 illustrate the adsorption isotherms and the calculated parameters. The correlation coefficient (R^2^) and the Akaike’s information criterion (AIC) were used to confirm the goodness of fit. Lower AIC values imply that the respective model is more likely than the alternative model to characterize the sorption process [37]. Based on these parameters, it is clear that the Langmuir model fits better the experimental data for MB adsorption, whereas the Freundlich model describes better the adsorption process of MG at equilibrium. The favorability of the adsorption process was estimated from the values of the separation factor (*R_L_*), using the following equation:

(7) $$R_{L}=\frac{1}{1+K_{L}C_{0}}$$

where *C*_0_ (mg L^−1^) is the initial dye concentration, and *K_L_* is the Langmuir equilibrium constant.

When *R_L_* is between 0 and 1 the adsorption process is considered as favorable, for *R_L_* > 1 the adsorption is unfavorable, while for *R_L_* = 1 the process is linear [38]. In this study, the calculated values of *R_L_* fall between 0 and 1, which shows the favorability of the adsorption process.

The modified Langmuir isotherm model can be used to determine the competitive adsorption capacity of dyes in binary solutions [39]. Equation (8) describes this model mathematically.

(8) $$Q_{e,D1}=\frac{Q_{max,D1}K_{L,D1}C_{e,D1}}{1+K_{L,D1}C_{e,D1}+K_{L,D2}C_{e,D2}}$$

The linearized form of Equation (8) is the following:

(9) $$\frac{1}{Q_{e,D1}}=\frac{1}{Q_{max,D1}}+\frac{1}{Q_{max,D1}K_{L,D1}}\;\left[\frac{1}{C_{e,D1}}+\frac{K_{L,D2}C_{e,D2}}{C_{e,D1}}\right]$$

For dye 2 (*D*2), the linearized equation is:

(10) $$\frac{1}{Q_{e,D2}}=\frac{1}{Q_{max,D2}}+\frac{1}{Q_{max,D2}K_{L,D2}}\;\left[\frac{1}{C_{e,D2}}+\frac{K_{L,D1}C_{e,D1}}{C_{e,D2}}\right]$$

where *C_e_*_,*D*1_, *C_e_*_,*D*2_, *Q_e_*_,*D*1_, and *Q_e_*_,*D*2_ are the equilibrium concentration and the equilibrium adsorption capacity of dye 1 (*D*1) and dye 2 (*D*2) in binary solutions; *K_L_*_,*D*1_ and *K_L_*_,*D*2_ are the Langmuir constants characteristics for the dye adsorption from single solutions; and *Q_max_*_,*D*1_ and *Q_max_*_,*D*2_ are the maximum adsorption capacities of the magnetic composite for *D*1 and *D*2 in binary solution.

*Q_max_*_,*D*1_ and *Q_max_*_,*D*2_ can be obtained by graphing Equations (9) and (10), respectively. The ratio $\frac{Q_{max,binary}}{Q_{max,single}}\;$ offers information about the dynamics of dye adsorption in binary solutions [40]. The two adsorbates have a synergistic effect when this ratio is supraunitary, with the mixture’s effect stronger than the individual adsorbates’ effect. When the ratio is less than 1, the two adsorbates have an antagonistic effect, with the mixture having a weaker effect than the individual adsorbates. When the ratio is equal to 1, the combination has no influence on the dyes’ adsorption [40]. In Table 6, the values of *Q_max,MB_* and *Q_max,MG_*, as well as the ratio *Q_max,binary_*/*Q_max,single_* for each magnetic nanocomposite investigated in this study, are displayed. Only in the case of MB adsorption onto Fe_3_O_4_-SBA15 is the *Q_max,binary_*/*Q_max,single_* ratio equal to 1, implying that the mixture has no influence on the adsorption of each adsorbate. In all other cases, the ratio is subunitary, which means that the adsorption of each dye is hindered by the presence of the other one.

#### 3.2.5. Adsorption Mechanism

**Figure 17:**
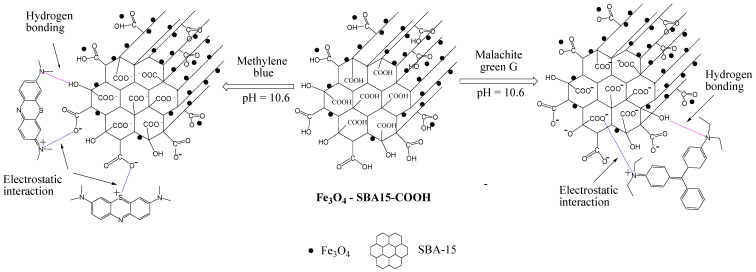
Schematic illustration of the possible adsorption mechanism of MB and MG onto carboxyl-functionalized magnetic nanocomposites through hydrogen bondings or electrostatic interactions.

In general, a high adsorption capacity is linked to a large surface area and pore volume of the adsorbent. For the magnetic nanocomposites investigated in this study, these two parameters have close values (Table 1); therefore, the differences between their adsorption capacities depend on other factors, such as the size of the adsorbate molecule and existing functional groups that may cause weaker or stronger interactions with the adsorbent surface. The structure and surface chemistry of the adsorbent also play a major role in dye molecule adsorption. During the adsorption process, a variety of interactions could occur such as hydrogen bonding, electrostatic interactions, van der Waals forces, and π-π interactions [41]. The driving force of adsorption, according to some researchers, is the molecular geometry and surface charge [42]. According to zeta potential measurements, the magnetic nanocomposites investigated in our study have negative charges (−32.4 mV for Fe_3_O_4_-SBA15, −38.5 mV for Fe_3_O_4_-SBA15-COOH-0.05, and −45.8 mV for Fe_3_O_4_-SBA15-COOH-0.1), as expected, due to the presence of carboxyl and hydroxyl groups on their surface (Table 1). It can be noted that zeta potential values increase as the content of the COOH groups increases. These negative charges indicate that these nanocomposites present more favorable adsorption for cationic dyes and a very good dispersibility in water, which is very important for the adsorption of pollutants from aqueous solutions. The electrostatic interaction seems to be the dominant mechanism of adsorption, but the hydrogen bonding could also be involved in the adsorption process. A schematic representation of these interactions is shown in Figure 17. The analysis of equilibrium data showed that MB dye is better adsorbed by the studied magnetic nanocomposites than MG dye. These experimental results could be explained by the molecular geometry of the dye molecule. The MB molecule, having a planar structure, favors the adsorption via a face-to-face conformation. On the other hand, nonplanar molecules such as MG are kept aside from the adsorbent surface due to the spatial constraint, resulting in low interactions with the adsorbent surface. This behavior was also observed by other researchers who investigated dye adsorption by modified multiwalled carbon nanotubes. They found that molecules with a greater charge and planar structure have a higher adsorption affinity [42].

#### 3.2.6. Comparison with Other Adsorbents

Table 7 shows a comparison of the performance of the magnetic nanocomposites investigated in this study with other similar adsorbents reported in the literature. As can be seen, their adsorption capacity is comparable to or even superior to that of the other adsorbents. Therefore, these novel magnetic nanocomposites might be considered as effective adsorbents to remove cationic dyes and other contaminants from wastewater.

#### 3.2.7. Desorption Studies

**Figure 18:**
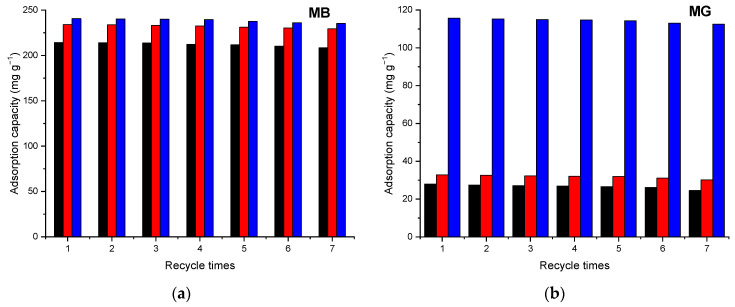
Reusability of the samples Fe_3_O_4_-SBA15 (black), Fe_3_O_4_-SBA15-COOH-0.05 (red), and Fe_3_O_4_-SBA15-COOH-0.1 (blue) for MB (**a**) and MG (**b**).

The desorption and regeneration capacity of the adsorbents are of crucial importance when assessing their industrial applications. In our study ethanol 94% has been tested as desorbing agent. As can be seen in Figure 18, after seven repeated adsorption–desorption cycles, the adsorption capacity of the adsorbents exhibited insignificant decay. Therefore, the nanocomposites exhibited superior recycling stability for the removal of organic dyes MB and MG from aqueous solutions.

## 4. Conclusions

In this study, three novel magnetic nanocomposites based on carboxyl-functionalized SBA-15 silica and magnetite nanoparticles, with relatively high surface area and total pore volume, were prepared through a simple method, and their adsorption capacity for methylene blue and malachite green G from single and binary aqueous solutions was investigated comparatively. An increase in the sorption capacity for both dyes by increasing the amount of carboxyl groups on the adsorbent surface was observed. The results showed that a pH value of 10.6 is the most favorable for dye adsorption. The kinetic studies revealed that dye adsorption onto all three adsorbents followed a pseudo-second-order kinetics model, the electrostatic interaction being the dominant mechanism of adsorption. The hydrogen bonding seems to be also involved in the adsorption process. The results of the intraparticle diffusion model indicated that the adsorption process is significantly influenced by external mass transfer and intraparticle diffusion. The equilibrium adsorption data recorded for MB were best fitted by the Langmuir model, whereas for MG, the Freundlich model described better the adsorption process at equilibrium. The maximum adsorption capacities were determined to be 239.17/30.73 mg g^−1^ (MB/MG) for Fe_3_O_4_-SBA15, 254.58/39.28 mg g^−1^ (MB/MG) for Fe_3_O_4_-SBA15-COOH-0.05, and 256.09/126.55 mg g^−1^ (MB/MG) for Fe_3_O_4_-SBA15-COOH-0.1. These values are higher than most of those reported in the literature for similar materials. The obtained nanocomposites proved to be very good adsorbents for cationic dyes in single and binary solutions. Their high recycling stability, regeneration capacity, and efficient reuse in multiple cycles, recommends these nanocomposites as promising materials for wastewater treatment.

## Figures and Tables

**Table 1:** Textural parameters (S_BET_, total pore volume, average pore size) of the samples.

Sample	S_BET_ (m^2^ g^−1^)	Total Pore Volume (cm^3^ g^−1^)	Average Pore Size (nm)
SBA-15	689.9	1.333	7.1
SBA15-COOH-0.05	581.3	1.060	6.4
SBA15-COOH-0.1	559.3	1.294	8.4
Fe_3_O_4_-SBA15	442.1	1.072	8.4
Fe_3_O_4_-SBA15-COOH-0.05	471.1	1.025	7.7
Fe_3_O_4_-SBA15-COOH-0.1	469.0	1.032	8.5

**Table 2:** The kinetic parameters for dye adsorption onto magnetic nanocomposites from single solutions (nonlinear regression).

Sample	Fe_3_O_4_-SBA15	Fe_3_O_4_-SBA15-COOH-0.05	Fe_3_O_4_-SBA15-COOH-0.1
**MB**			
*Q_e exp_* (mg g^−1^)	214.20	234.04	240.60
Pseudo-first-order model			
*Q_e cal_* (mg g^−1^)	202.03 ± 5.90	226.58 ± 5.23	230.96 ± 6.37
*k*_1_ (min^−1^)	0.0636 ± 0.0080	0.0607 ± 0.0060	0.0699 ± 0.0085
R^2^_adjusted_	0.9587	0.9744	0.9606
**Pseudo-second-order model**			
*Q_e cal_* (mg g^−1^)	217.89 ± 3.06	243.46 ± 1.94	246.98 ± 2.96
*k*_2_ (10^−4^ g mg^−1^ min^−1^)	4.0081 ± 0.3314	3.5282 ± 0.1651	4.0973 ± 0.2987
R^2^_adjusted_	0.9930	0.9977	0.9944
**MG**			
*Q_e exp_* (mg g^−1^)	27.92	32.8	115.64
**Pseudo-first-order model**			
*Q_e cal_* (mg g^−1^)	27.61 ± 1.34	30.89 ± 1.74	107.14 ± 4.02
*k*_1_ (min^−1^)	0.0108 ± 0.0016	0.0152 ± 0.0030	0.0200 ± 0.0028
R^2^_adjusted_	0.9731	0.9390	0.9658
**Pseudo-second-order model**			
*Q_e cal_* (mg g^−1^)	34.40 ± 1.80	36.43 ± 2.06	125.59 ± 4.55
*k*_2_ (10^−4^ g mg^−1^min ^−1^)	3.2858 ± 0.6712	5.0231 ± 1.2543	1.8146 ± 0.3050
R^2^_adjusted_	0.9856	0.9694	0.9843

**Table 3:** The kinetic parameters for dye adsorption onto magnetic nanocomposites from binary solutions (nonlinear regression).

Sample	Fe_3_O_4_-SBA15	Fe_3_O_4_-SBA15-COOH-0.05	Fe_3_O_4_-SBA15-COOH-0.1
**MB (MB + MG)**			
*Q_e exp_* (mg g^−1^)	174.48	193.16	201.84
**Pseudo-first-order model**			
*Q_e cal_* (mg g^−1^)	168.43 ± 3.90	188.33 ± 2.79	196.20 ± 2.59
*k*_1_ (min^−1^)	0.0463 ± 0.0045	0.0747 ± 0.0049	0.1059 ± 0.0067
R^2^_adjusted_	0.9780	0.9884	0.9893
**Pseudo-second-order model**			
*Q_e cal_* (mg g^−1^)	183.91 ± 1.53	200.34 ± 1.79	206.57 ± 1.64
*k*_2_ (10^−4^ g mg^−1^ min^−1^)	3.3498 ± 0.1546	5.5057 ± 0.3057	8.0765 ± 0.4467
R^2^_adjusted_	0.9980	0.9968	0.9969
**MG (MB + MG)**			
*Q_e exp_* (mg g^−1^)	23.24	27.32	89.80
**Pseudo-first-order model**			
*Q_e cal_* (mg g^−1^)	24.09 ± 0.50	27.20 ± 1.19	88.82 ± 2.18
*k*_1_ (min^−1^)	0.0111 ± 0.0072	0.0136 ± 0.0020	0.0178 ± 0.0016
R^2^_adjusted_	0.9951	0.9687	0.9878
**Pseudo-second-order model**			
*Q_e cal_* (mg g^−1^)	30.62 ± 0.20	32.76 ± 1.58	105.70 ± 3.35
*k*_2_ (10^−4^ g mg^−1^ min^−1^)	3.5855 ± 0.0981	4.6580 ± 0.9505	1.8420 ± 0.2619
R^2^_adjusted_	0.9952	0.9821	0.9905

**Table 4:** Kinetic parameters obtained from the fitting of the experimental data with intraparticle diffusion model.

Dye	Sample	Breakpoint (min^1/2^)	*k_id_* (mg g^−1^ min^−1/2^)	*C* (mg g^−1^)	R^2^
MB	Fe_3_O_4_-SBA15	4.8	31.36	2.59	0.9998
		14.7	6.06	124.70	0.9936
	Fe_3_O_4_-SBA15-COOH-0.05	6.2	27.99	21.50	0.9952
		13.5	5.31	162.12	0.9659
	Fe_3_O_4_-SBA15-COOH-0.1	4.9	30.66	26.54	0.9954
		12.4	8.27	137.28	0.9758
MG	Fe_3_O_4_-SBA15	-	2.21	−4.88	0.9865
	Fe_3_O_4_-SBA15-COOH-0.05	-	2.45	−3.21	0.9809
	Fe_3_O_4_-SBA15-COOH-0.1	-	8.59	−8.14	0.9550

**Table 5:** Langmuir and Freundlich isotherm parameters for adsorption of MB and MG onto magnetic nanocomposites.

Sample	Fe_3_O_4_-SBA15	Fe_3_O_4_-SBA15-COOH-0.05	Fe_3_O_4_-SBA15-COOH-0.1
**Dye**	**MB**		
Langmuir parameters			
*Q_max_* (mg g^−1^)	239.17	254.58	256.09
*K_L_* (L mg^−1^)	0.2544	0.2574	0.3157
R^2^	0.9928	0.9964	09966
AIC	39.39	35.87	35.83
*R_L_*	0.440	0.437	0.387
Freundlich parameters			
*K_F_* (mg g^−1^)	65.33	68.02	74.89
1/*n*	0.3382	0.3523	0.3372
R^2^	0.8830	0.9244	0.9375
AIC	56.14	54.24	53.42
**Dye**	**MG**		
Langmuir parameters			
*Q_max_* (mg g^−1^)	30.73	39.28	126.55
*K_L_* (L mg^−1^)	0.0531	0.0423	0.0234
R^2^	0.9502	0.9848	0.9934
AIC	22.82	18.62	25.50
*R_L_*	0.790	0.825	0.895
Freundlich parameters			
*K_F_* (mg g^−1^)	4.11	4.16	6.41
1/*n*	0.4167	0.4588	0.5977
R^2^	0.9833	0.9866	0.9975
AIC	16.24	17.87	19.58

**Table 6:** The maximum adsorption capacities determined using the modified Langmuir isotherm model in binary solutions.

Adsorbent	Dye	Parameters	Binary Solution (mg g^−1^)	$\frac{Q_{max,binary}}{Q_{max,single}}$
Fe_3_O_4_-SBA15	MB	*Q_max,MB_*	239.23	1.00
	MG	*Q_max,MG_*	24.33	0.79
Fe_3_O_4_-SBA15-COOH-0.05	MB	*Q_max,MB_*	181.48	0.71
	MG	*Q_max,MG_*	36.58	0.93
Fe_3_O_4_-SBA15-COOH-0.1	MB	*Q_max,MB_*	154.32	0.60
	MG	*Q_max,MG_*	81.10	0.23

**Table 7:** Comparison of adsorption capacity of various adsorbents for MB and MG.

Adsorbent	Adsorption Capacity for MB (mg g^−1^)	Adsorption Capacity for MG (mg g^−1^)	Reference
Fe_3_O_4_@SiO_2_-EDA-COOH	43.15	-	[43]
γ-Fe_2_O_3_/SiO_2_ nanocomposite	116.10	-	[44]
Mesoporous Fe_3_O_4_@SiO_2_	33.12	-	[45]
Fe_3_O_4_@SiO_2_-CR	31.44	-	[46]
Fe_2_O_3_@mSiO_2_	208.31	-	[47]
Carboxylic-functionalized superparamagnetic mesoporous silica microspheres	109.80	-	[20]
CoFe_2_O_4_-SiO_2_	-	75.50	[48]
Fe_3_O_4_@SiO_2_-CPTS magnetic NPs	-	25.50	[49]
GO	-	27.16	[50]
SWCNT-COOH	-	19.84	[51]
Fe_3_O_4_-SBA15	239.17	30.73	This work
Fe_3_O_4_-SBA15-COOH-0.05	254.58	39.28	This work
Fe_3_O_4_-SBA15-COOH-0.1	256.09	126.55	This work

## Supplementary Materials

The following supporting information can be downloaded at: www.mdpi.com/article/10.3390/nano12132247/s1, Figure S1: FTIR spectra of SBA15, SBA15-COOH-0.05 and SBA15-COOH-0.1; Figure S2: Nitrogen adsorption–desorption isotherms (left) and pore size distribution curves (right) of SBA15, SBA15-COOH-0.05, and SBA15-COOH-0.1; Figure S3: Equilibrium adsorption isotherms of MB on the magnetic adsorbents at pH = 10.6 and 298 K; Figure S4: Equilibrium adsorption isotherms of MG on the magnetic adsorbents at pH = 10.6 and 298 K.
