# Supplementary material for: Novel Magnetic Nanocomposites Based on Carboxyl-Functionalized SBA-15 Silica for Effective Dye Adsorption from Aqueous Solutions
Source: Nanomaterials (Basel). 2022 Jun 29;12(13):2247. doi: 10.3390/nano12132247 (PMC9268668; doi:10.3390/nano12132247)
Supplement: Supplementary file 1 [file nanomaterials-12-02247-s001.zip › nanomaterials-1784361-supplementary.pdf]

nanomaterials

Supplementary Materials

Novel Magnetic Nanocomposites Based on Carboxyl-Functionalized SBA-15 Silica for Effective Dye Adsorption from Aqueous Solutions

Claudia Maria Simonescu [1], Daniela Cristina Culita [2,*], Alina Tatarus [1], Teodora Mocanu [2], Gabriela Marinescu [2], Raul Augustin Mitran [2], Irina Atkinson [2], Andrei Kuncser [3] and Nicolae Stanica [2]

[1] Faculty of Chemical Engineering and Biotechnologies, Politehnica University of Bucharest, Polizu Street, No.1-7, 011061 Bucharest, Romania; claudia.simonescu@upb.ro (C.M.S.); alina.tatarus@yahoo.com (A.T.)
[2] Ilie Murgulescu Institute of Physical Chemistry, Romanian Academy, 202 Splaiul Independentei, 060021 Bucharest, Romania; tmocanu@icf.ro (T.M.); gmarinescu@icf.ro (G.M.); rmitran@icf.ro (R.A.M.); iatkinson@icf.ro (I.A.); nstanica@icf.ro (N.S.)
[3] National Institute of Materials Physics, Atomistilor Street No. 405 A, 077125 Magurele, Romania; andrei.kuncser@infim.ro
* Correspondence: dculita@icf.ro or danaculita@yahoo.co.uk; Tel.: +40-213167912

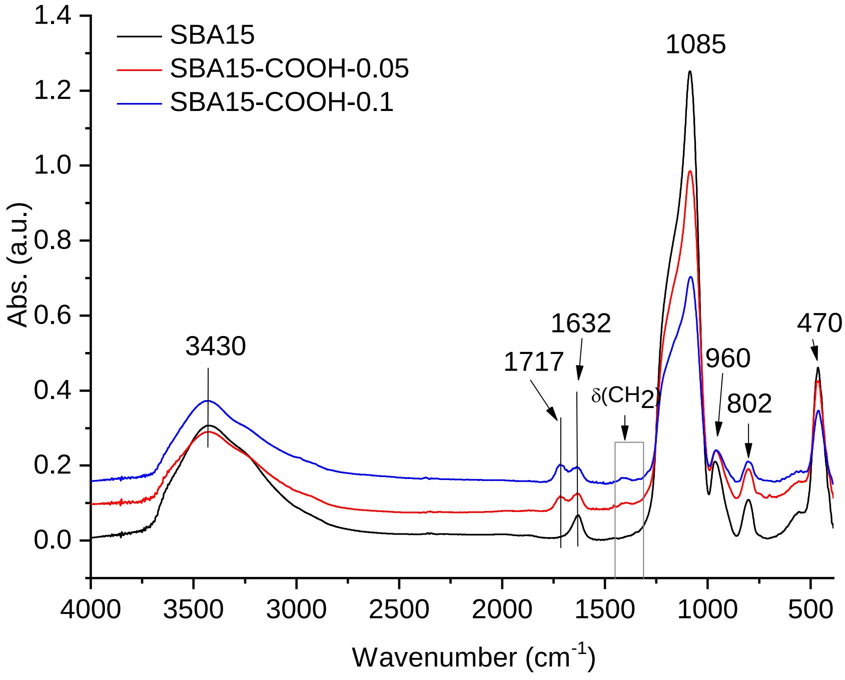

Figure S1. FTIR spectra of SBA15, SBA15-COOH-0.05 and SBA15-COOH-0.1.

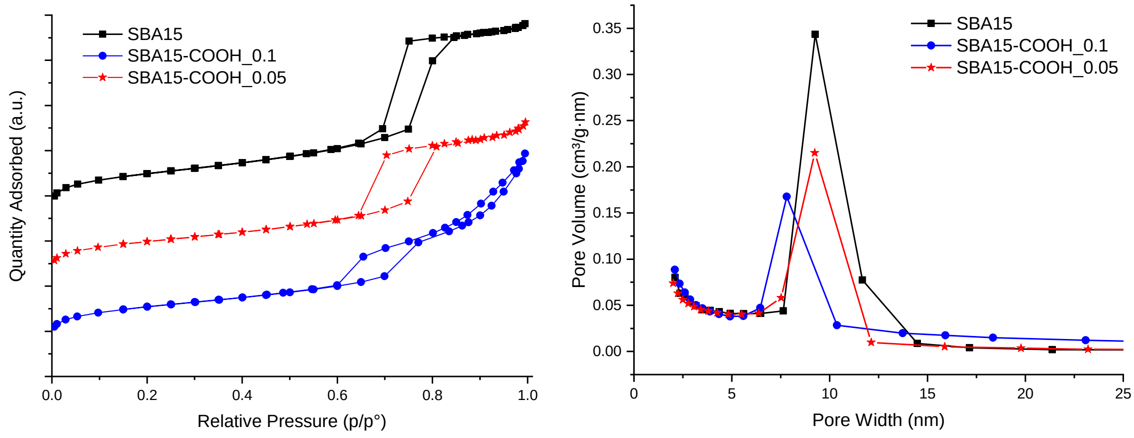

Figure S2. Nitrogen adsorption–desorption isotherms (left) and pore size distribution curves (right) of SBA15, SBA15-COOH-0.05, and SBA15-COOH-0.1.

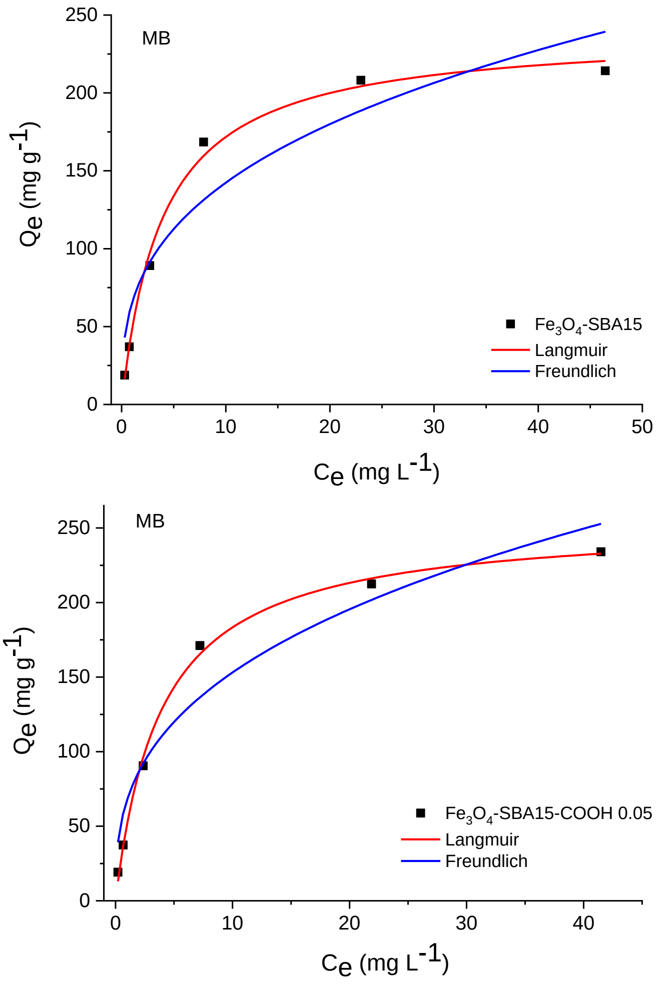

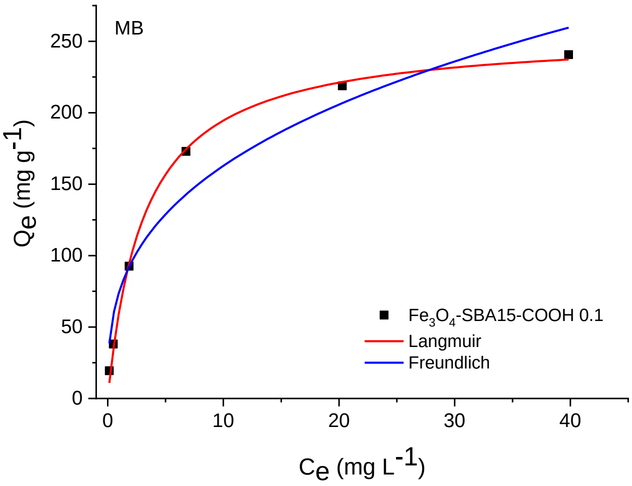

Figure S3. Equilibrium adsorption isotherms of MB on the magnetic adsorbents at pH = 10.6 and 298 K.

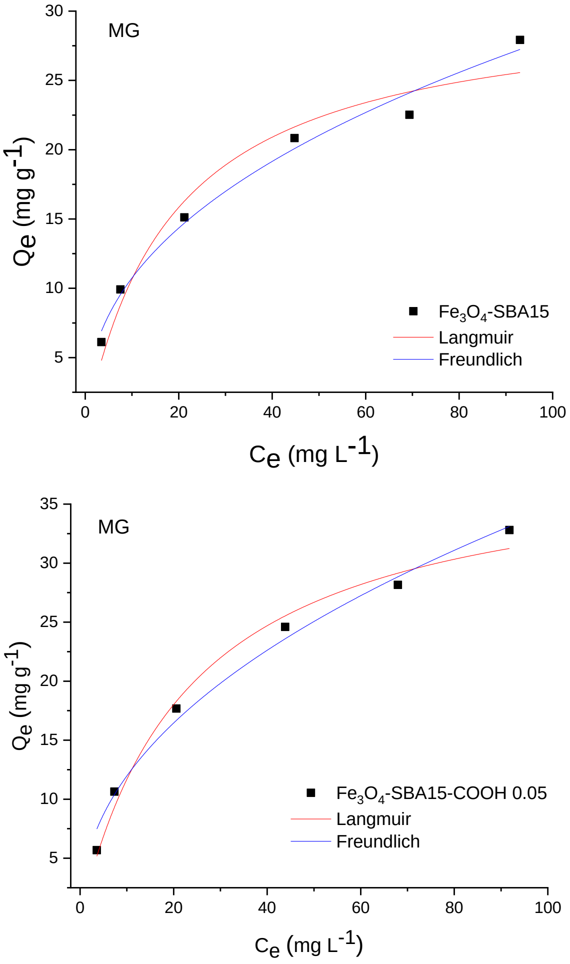

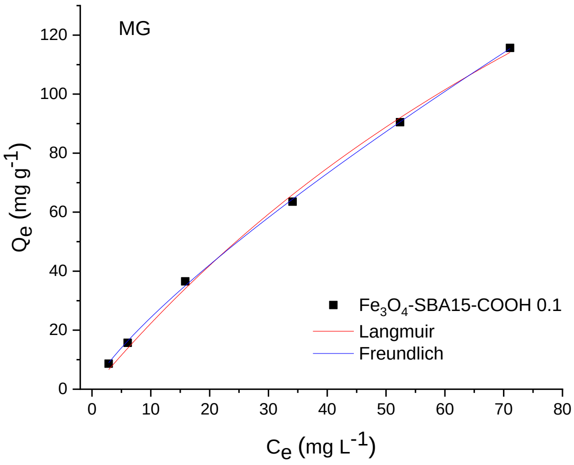

Figure S4. Equilibrium adsorption isotherms of MG on the magnetic adsorbents at pH = 10.6 and 298 K.
